# The Challenges of Vaccine Trial Participation among Underserved and Hard-to-Reach Communities: An Internal Expert Consultation of the VACCELERATE Consortium

**DOI:** 10.3390/vaccines11121784

**Published:** 2023-11-29

**Authors:** Dimitrios Poulimeneas, Markela Koniordou, Dimitra Kousi, Christina Merakou, Ioannis Kopsidas, Grammatiki Christina Tsopela, Christos D. Argyropoulos, Sophia C. Themistocleous, George Shiamakkides, Marinos Constantinou, Alexandra Alexandrou, Evgenia Noula, Andria Nearchou, Jon Salmanton-García, Fiona A. Stewart, Sarah Heringer, Kerstin Albus, Elena Álvarez-Barco, Alan Macken, Romina Di Marzo, Catarina Luis, Paula Valle-Simón, Helena H. Askling, Margot Hellemans, Orly Spivak, Ruth Joanna Davis, Anna Maria Azzini, Imre Barta, Lenka Součková, Ligita Jancoriene, Murat Akova, Patrick W. G. Mallon, Ole F. Olesen, Jesus Frias-Iniesta, Pierre van Damme, Krisztina Tóth, Miriam Cohen-Kandli, Rebecca Jane Cox, Petr Husa, Pontus Nauclér, Laura Marques, Jordi Ochando, Evelina Tacconelli, Markus Zeitlinger, Oliver A. Cornely, Zoi Dorothea Pana, Theoklis E. Zaoutis

**Affiliations:** 1 Collaborative Center for Clinical Epidemiology and Outcomes Research (CLEO), 15451 Athens, Greece; d.poulimeneas@cleoresearch.org (D.P.); m.koniordou@gmail.com (M.K.); d.kousi@cleoresearch.org (D.K.); christina.merakou@gmail.com (C.M.); c.tsopela@cleoresearch.org (G.C.T.); 2 School of Medicine, European University Cyprus, Nicosia 2404, Cyprus; c.argyropoulos@research.euc.ac.cy (C.D.A.); s.themistocleous@euc.ac.cy (S.C.T.); mconst24@gmail.com (M.C.); a.alexandrou91@gmail.com (A.A.); e.noula@research.euc.ac.cy (E.N.); a.nearchou@research.euc.ac.cy (A.N.); z.pana@euc.ac.cy (Z.D.P.); 3 Cologne Excellence Cluster on Cellular Stress Responses in Aging-Associated Diseases (CECAD), Institute of Translational Research, Faculty of Medicine and University Hospital Cologne, University of Cologne, 50931 Cologne, Germany; jon.salmanton-garcia@uk-koeln.de (J.S.-G.); sarah.heringer@uk-koeln.de (S.H.); oliver.cornely@uk-koeln.de (O.A.C.); 4 German Centre for Infection Research (DZIF), Partner Site Bonn-Cologne, 38124 Cologne, Germany; 5 Center for Integrated Oncology Aachen Bonn Cologne Duesseldorf (CIO ABCD) and Excellence Center for Medical Mycology (ECMM), Department I of Internal Medicine, Faculty of Medicine and University Hospital Cologne, University of Cologne, 50931 Cologne, Germany; 6 Centre for Experimental Pathogen Host Research, School of Medicine, University College Dublin, D04 V1W8 Dublin, Ireland; elena.alvarezbarco@ucd.ie (E.Á.-B.); alan.macken@ucd.ie (A.M.); paddy.mallon@ucd.ie (P.W.G.M.); 7 European Vaccine Initiative (EVI), 69115 Heidelberg, Germany; romina.dimarzo@euvaccine.eu (R.D.M.); catarina.luis@euvaccine.eu (C.L.); ole.olesen@euvaccine.eu (O.F.O.); 8 Hospital La Paz Institute for Health Research (IdiPAZ), 28046 Madrid, Spain; paula.ucicec@gmail.com (P.V.-S.); jesus.frias@uam.es (J.F.-I.); 9 Servicio Madrileño de Salud, 28046 Madrid, Spain; 10 Department of Infectious Diseases, Karolinska University Hospital, 17177 Stockholm, Sweden; helena.hervius.askling@ki.se (H.H.A.); pontus.naucler@ki.se (P.N.); 11 Department of Medicine, Division of Infectious Diseases, Karolinska Institutet, 17177 Stockholm, Sweden; 12 VAXINFECTIO, Centre of Evaluation of Vaccination, Faculty of Medicine and Health Science, Universiteit Antwerpen, 2610 Antwerp, Belgium; margot.hellemans@uantwerpen.be (M.H.); pierre.vandamme@uantwerpen.be (P.v.D.); 13 Ministry of Health of Israel, Jerusalem 1176, Israel; orlee.f@gmail.com (O.S.); miriam.cohen-k@moh.gov.il (M.C.-K.); 14 Infectious Diseases, Department of Diagnostic and Public Health, University of Verona, 37134 Verona, Italy; ruthjoanna.davis@univr.it (R.J.D.); annamaria.azzini@univr.it (A.M.A.); evelina.tacconelli@univr.it (E.T.); 15 National Koranyi Institute for Pulmonology, 1121 Budapest, Hungarytoth.k@koranyi.hu (K.T.); 16 Department of Pharmacology, Faculty of Medicine, Masaryk University, 60177 Brno, Czech Republic; lsouckova@med.muni.cz (L.S.); husa.petr@fnbrno.cz (P.H.); 17 University Hospital Brno, 62500 Brno, Czech Republic; 18 Czech Clinical Research Infrastructure Network (CZECRIN), 62500 Brno, Czech Republic; 19 Institute of Clinical Medicine, Medical Faculty, Vilnius University, 03101 Vilnius, Lithuania; ligita.jancoriene@santa.lt; 20 Vilnius University Hospital Santaros Klinikos, Medical Faculty, Vilnius University, 03101 Vilnius, Lithuania; 21 Department of Infectious Diseases and Clinical Microbiology, Faculty of Medicine, Haceteppe University, Ankara 06230, Turkey; makova@hacettepe.edu.tr; 22 Influenza Centre, Department of Clinical Science, University of Bergen, 5020 Bergen, Norway; rebecca.cox@uib.no; 23 Centro Hospitalar Universitário do Porto, 4099-001 Porto, Portugal; laurahoramarques@gmail.com; 24 Centro Nacional de Microbiología, Instituto de Salud Carlos III, 28220 Madrid, Spain; jochando@isciii.es; 25 Department of Clinical Pharmacology, Medical University Vienna, 1090 Vienna, Austria; markus.zeitlinger@meduniwien.ac.at; 26 Clinical Trials Centre Cologne (ZKS Köln), Faculty of Medicine and University Hospital Cologne, University of Cologne, 50935 Cologne, Germany; 27 Center for Molecular Medicine Cologne (CMMC), Faculty of Medicine and University Hospital Cologne, University of Cologne, 50931 Cologne, Germany; 28 2nd Department of Pediatrics, School of Medicine, National and Kapodistrian University of Athens, 11527 Athens, Greece

**Keywords:** barriers, COVID-19, Europe, pandemic preparedness, SARS-CoV-2, vaccinations, vaccine education, vaccine trials

## Abstract

Underserved and hard-to-reach population groups are under-represented in vaccine trials. Thus, we aimed to identify the challenges of vaccine trial participation of these groups in member countries of the VACCELERATE network. Seventeen National Coordinators (NC), each representing their respective country (15 European countries, Israel, and Turkey), completed an online survey. From 15 eligible groups, those that were more frequently declared underserved/hard-to-reach in vaccine research were ethnic minorities (76.5%), persons experiencing homelessness (70.6%), illegal workers and refugees (64.7%, each). When prioritization for education on vaccine trials was considered, ethnic groups, migrants, and immigrants (5/17, 29.4%) were the groups most frequently identified by the NC as top targets. The most prominent barriers in vaccine trial participation affecting all groups were low levels of health literacy, reluctance to participate in trials due to engagement level, and low levels of trust in vaccines/vaccinations. This study highlighted population groups considered underserved/hard-to-reach in countries contained within the European region, and the respective barriers these groups face when participating in clinical studies. Our findings aid with the design of tailored interventions (within—and across—countries of the European region) and with the development of strategies to overcome major barriers in phase 2 and phase 3 vaccine trial participation.

## 1. Introduction

The SARS-CoV-2 pandemic has reshaped clinical research by enhancing the growth of vaccine trials at unprecedented rates [1]. Despite this, published prevention and treatment clinical trials have failed to represent diverse societal groups in phase 2 and 3 vaccine trials [2], including those that can be characterized as “underserved” or “hard-to-reach” (HTR). These terms are commonly used to describe population groups that have inadequate access to healthcare for any reason, including, but not limited to, dislocated populations, persons of unknown legal status, and social minorities [3]. How these groups differ biologically from other populations within the same country is debatable; however, published clinical trials report differences in the immunogenicity of vaccines among these groups against many infectious diseases (i.e., influenza, hepatitis B, and SARS-CoV-2 viruses) [4,5,6]. Therefore, the effectiveness of interventions and medical therapies can only be evaluated when research is inclusive of minorities and disadvantaged populations; otherwise, the scientific findings are limited to majority groups, and consequently, health disparities are further reinforced [7].

VACCELERATE (www.vaccelerate.eu, accessed on 21 November 2023) is a clinical research network that orchestrates the coordination and conduct of COVID-19 clinical trials. The network is currently active in 18 European Union (EU) countries and 5 countries associated with the European Commission’s activities for future pandemic preparedness, the Health Emergency Preparedness and Response Authority (HERA) Incubator, and the Horizon 2020 program. Its overarching goal is to accelerate vaccine research and enhance preparedness for the evolving SARS-CoV-2 pandemic, as well as those to come [8]. Each participating country is represented by its National Coordinator (NC), who is a professional/academic with extensive experience in communicable diseases, functioning as a country’s ambassador of the project. The core principles of the project are inclusion, diversity, and equity in clinical research [9,10].

It is of paramount importance to identify underserved/HTR groups in the dynamically demographically changing map of Europe so as to understand how to better engage them in future clinical studies [11,12,13], with the ultimate goal of addressing the health inequalities prevailing in the region. The aim of the survey was to identify the challenges of vaccine trial participation of underserved and HTR groups in countries that belong to the VACCELERATE consortium. Specific objectives were the following: (i) to identify underserved and HTR groups per participating country, (ii) to identify barriers to participation of these groups in vaccine clinical trials, and (iii) to identify the reasons stakeholders face difficulties in recruiting the above-mentioned groups in vaccine clinical trials.

## 2. Materials and Methods

**Figure 1:**
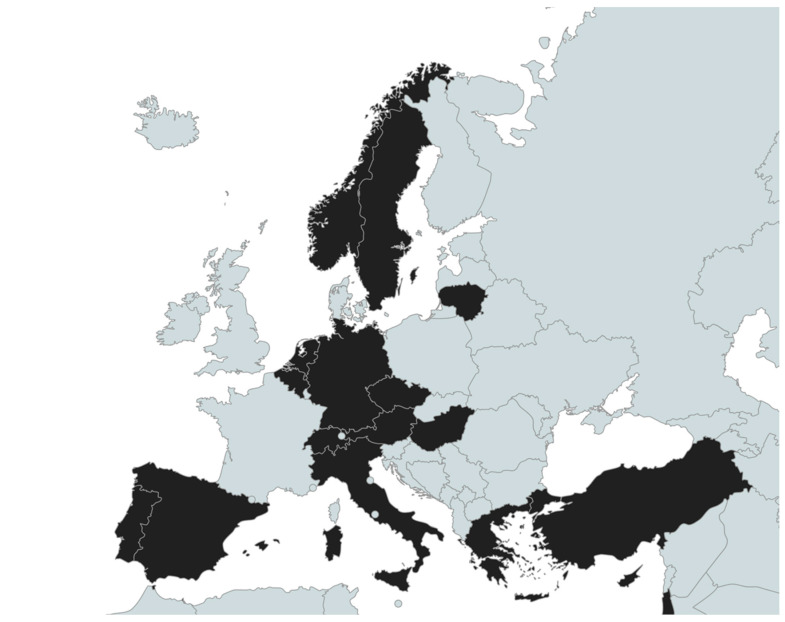
National coordination (NC) representatives from 17 countries (in black) took part in the survey between July and August 2021.

The original online survey was designed by members of the VACCELERATE consortium, based on literature relevant to the European context on underserved/HTR population groups [14,15,16,17]. The survey was developed using Google Forms (https://docs.google.com/forms/) (the full questionnaire is available upon request from the corresponding author) and was disseminated to all NC as part of an internal consultation between July and August 2021. The NC of the consortium are individuals with proven and extensive experience in infectious diseases, with most of them being faculty members of renowned academic institutions and/or members of major governmental and non-governmental entities (i.e., Ministries of Health, National Public Health Organizations, etc.). Seventeen VACCELERATE NC responded. Namely, the NC of Austria, Belgium, Cyprus, Czech Republic, Germany, Greece, Hungary, Israel, Italy, Lithuania, Netherlands, Norway, Portugal, Spain, Sweden, Switzerland, and Turkey. A schematic presentation of the countries participating in this survey is depicted in Figure 1.

### 2.1. Identification of Underserved/HTR Groups in Terms of Vaccine Trial Participation and Prioritization for Education on Vaccine Trials

A list of 15 population groups that are considered underserved/HTR was created, and the definition of each group was provided (please see Supplementary Table S1 for definitions). From these groups, the NC were asked to select the groups they considered to be underserved/HTR in vaccine clinical trials in their country (question: which of the following population groups do you consider as underserved and/or HTR communities in your country in terms of vaccine trial participation?) and the groups that they would prioritize for educating on vaccine trial participation (question: which of the above-chosen population groups would you prioritize for education purposes related to vaccine clinical trials). This list included the following: (a) ethnic minorities, (b) persons experiencing homelessness (PEH), (c) illegal workers, (d) refugees, (e) sex workers, (f) migrants, (g) immigrants, (h) religious groups, (i) chronic drug users, (j) geographically isolated groups, (k) children (<18 years old), (l) pregnant/lactating women, (m) LGBT+, (n) emigrants, and (o) older adults (>65 years old).

### 2.2. Barriers of Underserved/HTR Groups to Participating in Vaccine Clinical Trials

NC were asked to identify the factors that might be currently preventing the aforementioned underserved/HTR groups from participating in vaccine clinical trials in each country (question: what factors do you think may be currently preventing individuals from the selected communities from participating in vaccine clinical trials in your country?). These included (a) lack of access to health provider due to distance/time for traveling (i.e., the primary reason for inadequate access to healthcare is the large distance and/or time needed to visit a health provider); (b) lack of access to health provider due to geographic barriers, lack of transportation, etc. (i.e., the primary reason for inadequate access to healthcare is geographic isolation and/or lack of infrastructure to visit the health provider; (c) lack of access to health provider due to other reasons (socio-economic or legal) (i.e., the primary reasons for inadequate access to healthcare are socio-economic or legal reasons not described in the above-mentioned options (b) and (c)); (d) transient or nomadic movement; (e) lack of healthcare provider recommendations; (f) healthcare provider discrimination; (g) low levels of trust in healthcare; (h) low levels of trust in vaccines and/or vaccine production process; (i) low levels of health literacy; (j) compulsory vaccination schedule not provided for free; (k) war and/or local conflicts; (l) legal restrictions; (m) language barrier; (n) religious belief restrictions; (o) anonymity/privacy issues; (p) reluctance to participate in a vaccine trial due to engagement level; and (q) uncertainty regarding the impact on their health condition.

### 2.3. Reasons Why Stakeholders Have Difficulties in Recruiting Underserved/HTR Groups in Vaccine Clinical Trials

NC were asked to choose the reasons why stakeholders might find it challenging to reach underserved/HTR groups for vaccine trial recruitment from a list of 7 items. These included (a) lack of information adapted to the specific target group (e.g., language adaptation, selection of proper channel, etc.), (b) lack of communication channels (e.g., not enough advocacy groups, official media, etc.), (c) limited funds to actively recruit, (d) lack of national infrastructure, (e) lack of public health system access, (f) not a priority in national research and development agenda, and (g) legal status.

### 2.4. Data Analysis

Values are presented as absolute (n) or relative frequencies (%) of affirmative responses for each of the questions asked. Categorical variables are presented using frequency tables. Relative frequencies were calculated in Microsoft Excel. The map of the participating countries for Figure 1 was created through www.mapchart.net (accessed on 21 November 2023). 

## 3. Results

Identified underserved/HTR groups are summarized by country in Table 1. The three most frequently identified underserved/HTR groups were ethnic minorities (13/17, 76.5%), PEH (12/17, 70.6%), as well as illegal workers and refugees (11/17, 64.7% for both groups). On the contrary, the groups that were the least frequently considered as underserved/HTR were children (3/17, 17.6%), emigrants (2/17, 11.8%), and older adults (>65 years old) (n = 1/17, 5.9%). The countries that identified the highest number of groups as underserved/HTR were Belgium (10/15, 67.7%); Cyprus, Germany, and Sweden (9/15 for each country, 60.0%); and Israel, Norway, and Sweden (8/15 for each country, 53.3%). On the other hand, only 2 out of 15 groups were considered underserved/HTR in Hungary and Spain; 3 out of 15 in Austria; and 4 out of 15 in the Czech Republic, Portugal, and Switzerland.

Ethnic groups, migrants, and immigrants (5/17, 29.4%) were the groups that were most frequently identified as top targets for education on vaccine trial participation. In contrast, chronic drug users and older adults were not identified by any of the NC as being in need of prioritization (Table 2). A group-specific analysis of barriers to vaccine trial participation is depicted in Table 3. The groups that were found to be most affected by the majority of barriers were dislocated populations (refugees, immigrants, migrants, and emigrants) and ethnic minorities. What is more, refugees and illegal workers were found to be affected by all proposed barriers. To provide a more concise picture of the most pertinent barriers, we identified the barriers that affect at least 80% of the groups examined. The factors affecting all groups (100%) were low levels of health literacy, reluctance to participate in clinical trials due to engagement level, and low levels of trust in vaccines/vaccinations. Other key barriers were lack of healthcare provider recommendations (93.3%), access to health provider (distance/time for traveling) (80%), access to health provider (other reasons (social or legal)) (80%), uncertainty regarding the impact on their health condition (80%), and anonymity/privacy issues (80%). According to the collected data, lack of access to healthcare services mainly affects illegal workers and refugees (9/17, 52.9%), while “transient or nomadic movement” concerns PEH (7/17, 41.2%). Low levels of health literacy and language barriers are major barriers for ethnic groups. Language barriers were also considered a major barrier for refugees and immigrants (11/17, 64.7%), as well as migrants (10/17, 58.8%).

When asked to share the reasons why stakeholders might face difficulties in recruiting HTR groups in vaccine clinical trials, at least 50.0% of NC reported that a lack of information adapted to the specific target group affects ethnic minorities, PEH, refugees, migrants, and immigrants, while a lack of communication channels obstructs recruitment of PEH, refugees, and immigrants (Table 4).

## 4. Discussion

Clinical trial diversity and equity, through the participation of different population groups in clinical trials, is crucial in order to ensure the generalizability of medical research [18]. However, vaccine clinical trials suffer from inadequate representation of diverse population groups. The present internal consultation of the VACCELERATE consortium provided insight into which groups can be considered the most pertinent underserved/HTR groups in countries of the European region and Israel and identified possible barriers to vaccine clinical trial participation.

Ethnic minorities, PEH, and illegal workers were some of the groups that were most frequently considered underserved/HTR. Ethnic minorities have been consistently considered underrepresented in COVID-19 clinical trials, although they appear to be disproportionately affected by the disease [19]. Previous research has identified several barriers that may prevent the participation of ethnic minorities in clinical trials, such as skepticism of medical care and research, as well as language discordance [20]. PEH comprises a significant minority in Europe, with up to 5% of individuals in eight European countries having experienced homelessness at least once during their lifetime [21]. Even though homeless people suffer from higher mortality rates compared to the general population—largely attributed to infectious diseases, mental health issues, drug and alcohol abuse, etc.—they are generally excluded from vaccine clinical trials, as they are considered at high risk for attrition [22]. Regarding illegal workers, lack of legal status often impedes access to healthcare. Undocumented individuals delay or do not seek health provisions for fear of the penalties related to their missing legal status [23], along with the frequent absence of documentation required for registration to a clinical trial.

Misinformation and misconceptions around vaccine development and effectiveness largely contribute to the lack of vaccine confidence in minority populations. These communities are more likely to have firsthand experience with barriers to accessing healthcare and, therefore, have a higher level of mistrust of medicine and research [24]. When NC were asked to select which groups they would prioritize for education on vaccine clinical trials, ethnic groups, migrants, and immigrants were frequently selected. All three groups are known to be highly hesitant in terms of vaccination [2] while little or no public information is available for population groups that are typically underrepresented in vaccine clinical trials [25]. Concerning migrants and immigrants, both groups have been shown to have low vaccination coverage [26]. Thus, an educational intervention could be an important step toward rebuilding trust between them and the medical community [13,27]. The disparities in the answers of the NC between the groups they believe are underserved and the groups that they would prioritize for education may be explained by the fact that ethnic groups, migrants, and immigrants are easier to locate and deliver intervention to than PEH and illegal workers. Thus, we believe that this reflects a low-hanging-fruit approach in optimizing the conduct of vaccine clinical trials.

Regardless of the group examined, eight relevant barriers to vaccine trial participation were highlighted. Namely, low levels of health literacy, reluctancy to participate in clinical trials due to engagement level, low levels of trust in vaccines/vaccinations, lack of healthcare provider recommendations, limited access to a health provider due to distance/time for traveling or other reasons (social or legal), uncertainty regarding the clinical trials’ impact on their own health condition, and anonymity/privacy issues. Previous original works and systematic syntheses have highlighted that barriers to vaccine trial participation are nested in both the research system and in the communities [28], including mistrust of healthcare and lack of education in vaccines/vaccinations [29], as well as distrust of host country in the case of migrant populations [30]. When geographical distance is concerned, accessibility is known to determine vaccine uptake [11,31], and we speculate the same would apply to the much more time-demanding process of being enrolled in a clinical trial [32]. The language barrier was recognized by many NC as a major hindrance to the inclusion of ethnic groups, refugees, migrants, and immigrants in vaccine clinical trials. Non-native speakers often struggle to comprehend the clinical trial process and communicate with the research team, which contributes to their lack of trust in research, thus hampering vaccine trial participation [33]. A possible solution to overcome this obstacle would be the expansion of clinical research teams to include trained interpreters and have minorities represented in the research teams [34].

Lack of information tailored to the specific target group and lack of communication channels were the two most frequently reported difficulties in recruiting underserved/HTR groups in vaccine clinical trials. A recent systematic review of strategies to overcome barriers to clinical trial enrollment concluded that rigorous multilevel approaches targeting both the provider of the trial and the participant were more successful [28]. Besides what is already stated in the literature [32], it is possible that future studies in the European region can provide culturally adapted information and employ communication channels relevant to the population in question (i.e., healthcare providers that work closely with select underserved groups, recruitment of community leaders, etc.), which may achieve higher rates of inclusion and diversity in their population of volunteers. Moreover, clinical and public health agencies and stakeholders could actively create the conditions for more inclusive trial design and implementation in the EU. Toward this goal, systematic inclusion of patient and public involvement (PPI) before, during, and after research into vaccines in underrepresented groups is needed, as is specific guidance on the inclusion of different groups. A good example of the latter is the guidance offered in the US by the FDA on the inclusivity of ethnic groups. This guidance includes clear definitions of each ethnic group across the country, thus actively allowing for evidence-driven representation of each group in clinical research [35]. These approaches are instrumental in ensuring equitable participation in clinical trials, fostering transparency, and reinforcing the credibility of vaccine research within diverse populations.

Several limitations need to be mentioned. This survey consists of an internal expert consultation of a European consortium, and only one representative person from each participating country filled in the survey. Although the National Coordinators of the consortium are highly esteemed professionals with experience in communicable diseases, some degree of subjectivity and selection bias is to be anticipated. There is a lack of systematic information on the national frequency of underserved/HTR communities across Europe, and such records are widely dependent on within-country metrics. While the survey covered a wide range of underserved/HTR groups, underrepresentation of some groups cannot be ruled out (i.e., people with disabilities, pandemic denialists, etc.). Furthermore, some groups/barriers that are only pertinent within a country may have been under-examined. Nonetheless, while caution in the interpretation of our results is advised, this consultation provides a basis for extended research on country and region-specific aspects of vaccine research on underserved/HTR groups. Despite these limitations, we are confident that this analysis provides much-needed insight into clinical studies conducted in the European region, given the scarcity of relevant data for the European population, as well as the recent acceleration of vaccine development in Europe [6].

## 5. Conclusions

In summary, the present internal consultation of the VACCELERATE consortium has highlighted the most pertinent groups considered underserved/HTR in countries contained within the European region and the respective barriers these groups face when participating in clinical studies. These findings can aid with the design of tailored interventions (within—and across—countries of the European region), as well as with the development of strategies to overcome major barriers in phase 2 and phase 3 vaccine clinical trial participation within the studied population groups. Further studies aiming to validate and expand upon our observations are imperative for comprehensive understanding and application in future research and public health initiatives.

## Figures and Tables

**Table 1:** Population groups considered underserved/hard-to-reach in terms of vaccine trial participation per VACCELERATE participating country.

	Ethnic Minorities	PEH	Illegal Workers	Refugees	Sex Workers	Migrants	Immigrants	Religious Groups	Chronic Drug Users	PLW	GIP	Children	LGBT+	Emigrants	Older Adults	Total (out of 15)
Austria				•		•	•									3
Belgium	•	•	•	•	•	•	•			•			•	•		10
Cyprus	•	•		•	•	•	•	•					•	•		9
Czech Republic	•	•	•						•							4
Germany	•	•	•	•	•	•	•		•		•					9
Greece		•	•	•	•					•			•			6
Hungary	•	•														2
Israel	•	•	•	•	•			•		•		•				8
Italy	•	•	•			•					•	•			•	7
Lithuania	•	•	•		•				•		•					6
The Netherlands	•	•		•			•	•								5
Norway	•	•	•	•	•	•	•		•							8
Portugal	•			•		•						•				4
Spain			•					•								2
Sweden	•	•	•	•	•	•	•	•	•							9
Switzerland	•							•		•	•					4
Turkey			•	•	•		•		•		•		•			7
Total (out of 17)	13	12	11	11	9	8	8	6	6	4	5	3	4	2	1	

• denotes an affirmative response. PEH, persons experiencing homelessness; PLW, pregnant/lactating women; GIP, geographically isolated populations; LGBT+, Lesbian, Gay, Bisexual, Transgender, Queer or questioning, Intersex, Asexual, Pansexual, Kink (not limited to).

**Table 2:** Population groups selected to be prioritized for education on vaccine clinical trials per VACCELERATE participating country.

	Ethnic Minorities	PEH	Illegal Workers	Refugees	Sex Workers	Migrants	Immigrants	Religious Groups	Chronic Drug Users	PLW	GIP	Children	LGBT+	Emigrants	Older Adults	Total (out of 15)
Austria				•		•	•									3
Belgium	•					•	•			•				•		5
Cyprus				•	•	•										3
Czech Republic	•	•	•													3
Germany						•										1
Greece					•					•		•				3
Hungary	•															1
Israel																0
Italy		•									•	•				3
Lithuania	•															1
The Netherlands	•						•	•								3
Norway				•		•	•									3
Portugal																0
Spain																0
Sweden							•									1
Switzerland								•								1
Turkey				•	•								•			3
Total (out of 17)	5	2	1	4	3	5	5	2	0	2	1	2	1	1	0	

• denotes an affirmative response; PEH, persons experiencing homelessness; PLW, pregnant/lactating women; GIP, geographically isolated populations; LGBT+, Lesbian, Gay, Bisexual, Transgender, Queer or questioning, Intersex, Asexual, Pansexual, Kink (not limited to).

**Table 3:** Factors that may be preventing various underserved/hard-to-reach populations from participating in vaccine clinical trials across the 17 VACCELERATE participating countries.

	Refugees	Illegal Workers	Ethnic Minorities	Immigrants	Migrants	Emigrants	PEH	Religious Groups	Chronic Drug Users	GIP	Sex Workers	PLW	LGBT+	Children	Older Adults
Language barrier	11	7	9	11	10	8	2	2	0	0	1	0	1	0	0
Low levels of health literacy	6	4	9	5	6	6	5	4	3	2	2	3	1	1	1
Reluctance to participate in trials due to engagement level	7	6	7	5	6	4	4	1	2	2	3	2	1	1	1
Low levels of trust in healthcare	5	7	6	7	6	4	4	6	2	1	0	0	2	0	0
Low levels of trust in vaccines/vaccinations	5	4	6	5	5	3	3	6	2	1	1	5	1	2	2
Access to health provider: other reasons (social or legal)	9	9	4	7	5	3	5	1	3	1	1	0	0	0	1
Access to health provider: distance/time for traveling	3	5	5	4	5	3	1	0	2	6	0	2	0	1	2
Uncertainty regarding the impact on their health condition	5	2	6	4	3	3	1	4	2	2	0	2	0	2	0
Religious belief restrictions	5	3	7	5	4	2	1	6	0	0	0	0	0	1	0
Access to health provider: geographic barriers, transportation	3	4	2	2	3	2	4	0	3	6	0	0	0	0	2
Lack of healthcare provider recommendations	4	2	2	5	4	2	2	2	1	2	1	2	1	1	0
Transient or nomadic movement	5	3	2	2	2	3	7	0	1	1	2	0	0	0	0
Anonymity/Privacy issues	4	4	1	1	1	1	3	1	3	0	4	0	3	1	0
Healthcare provider discrimination	4	2	2	2	1	2	2	0	2	1	2	0	2	0	0
Legal restrictions	1	5	0	0	0	0	0	0	0	0	1	0	0	0	0
War and/or local conflicts	4	2	0	0	0	0	0	0	0	0	0	0	0	0	0
Compulsory vaccination schedule not provided for free	1	2	0	1	0	0	0	0	0	0	0	0	0	0	0

Values represent the sum of NC considering a given factor as a barrier for the specific population group; denser coloring suggests higher sum. PEH, persons experiencing homelessness; PLW, pregnant/lactating women; GIP, geographically isolated populations; LGBT+, Lesbian, Gay, Bisexual, Transgender, Queer or questioning, Intersex, Asexual, Pansexual, Kink (not limited to).

**Table 4:** Reasons why stakeholders face difficulties in recruiting underserved/hard-to-reach groups in vaccine trials across the 17 VACCELERATE participating countries.

	Refugees	Immigrants	PEH	Illegal Workers	Migrants	Ethnic Minorities	Emigrants	LGBT+	Chronic Drug Users	PLW	Sex Workers	GIP	Religious Groups	Children	Older Adults
Lack of information adapted to the specific target group	12	10	9	7	11	9	8	7	5	6	7	6	7	3	2
Lack of communication channels	10	9	9	7	8	8	6	7	7	6	7	5	6	2	2
Limited funds to actively recruit	8	8	6	7	7	5	7	6	6	5	5	5	4	3	3
Lack of national infrastructure	6	7	6	4	6	4	5	3	2	4	3	3	2	2	2
Not a priority in national research and development agenda	2	2	2	3	2	1	2	2	2	1	2	2	3	0	0
Lack of public health system access	3	2	3	5	1	3	1	0	1	1	0	2	0	1	1
Legal status	5	2	2	3	1	0	0	0	1	1	0	0	0	0	0

Values represent the sum of NC considering a given factor as a barrier for the specific population group; denser coloring suggests higher sum. PEH, persons experiencing homelessness; PLW, pregnant/lactating women; GIP, geographically isolated populations; LGBT+, Lesbian, Gay, Bisexual, Transgender, Queer or questioning, Intersex, Asexual, Pansexual, Kink (not limited to).

## Data Availability

Data supporting reported results are available after communication with the corresponding author.

## Supplementary Materials

The following supporting information can be downloaded at https://www.mdpi.com/article/10.3390/vaccines11121784/s1. Table S1: Definitions of Underserved/Hard-To-Reach populations utilized for the present survey.
