# Supplementary material for: The Challenges of Vaccine Trial Participation among Underserved and Hard-to-Reach Communities: An Internal Expert Consultation of the VACCELERATE Consortium
Source: Vaccines (Basel). 2023 Nov 29;11(12):1784. doi: 10.3390/vaccines11121784 (PMC10747264; doi:10.3390/vaccines11121784)
Supplement: Supplementary file 1 [file vaccines-11-01784-s001.zip › vaccines-2697093-supplementary.pdf]

Supplementary Table S1. Definitions of Underserved/Hard-To-Reach populations utilized for the present survey

Emigrants	In the global context, a resident (national or alien) departing or exiting from one state intending to remain abroad for a period exceeding one year.
Hard-To-Reach	Those sub-groups of the population that are difficult to reach or involve in research or public health programmes due to their physical and geographical location (e.g., in mountains, forests or deserts) or their social and economic situation.
Immigrants	In the global context, a non-resident (both national or alien) arriving in a state with the intention to remain for a period exceeding a year.
LGBT+	Lesbian, Gay, Bisexual, Transgender, Queer or questioning, Intersex, Asexual, Pansexual, Kink (not limited to).
Migrants	In the global context, a person who is outside the territory of the state of which they are nationals or citizens and who has resided in a foreign country for more than one year irrespective of the causes, voluntary or involuntary, and the means, regular or irregular, used to migrate.
Refugees	In the global context, either a person who, owing to a well-founded fear of persecution for reasons of race, religion, nationality, political opinion or membership of a particular social group, is outside the country of nationality and is unable or, owing to such fear, is unwilling to avail themselves of the protection of that country, or a stateless person, who, being outside of the country of former habitual residence for the same reasons as mentioned before, is unable or, owing to such fear, unwilling to return to it.
Underserved	Those populations that face barriers and challenges in accessing and using resources, due to geographic location, religion, sexual orientation, gendered-identity, racial, and ethnic populations. Underserved populations usually encounter unique challenges (such as language and cultural barriers, physical and/or cognitive ability, alienage status, or age).
